# 3-(4-Bromo­phenyl­sulfin­yl)-5-chloro-2-methyl-1-benzofuran

**DOI:** 10.1107/S1600536812025846

**Published:** 2012-06-13

**Authors:** Hong Dae Choi, Pil Ja Seo, Uk Lee

**Affiliations:** a Department of Chemistry, Dongeui University, San 24 Kaya-dong, Busanjin-gu, Busan 614-714, Republic of Korea; b Department of Chemistry, Pukyong National University, 599-1 Daeyeon 3-dong, Nam-gu, Busan 608-737, Republic of Korea

## Abstract

In the title compound, C_15_H_10_BrClO_2_S, the 4-bromo­phenyl ring makes a dihedral angle of 86.85 (6)° with the mean plane [mean deviation = 0.009 (2) Å] of the benzofuran fragment. In the crystal, mol­ecules are linked by slipped π–π inter­actions between the benzene and the furan rings of adjacent mol­ecules [centroid–centroid distance = 3.884 (2), inter­planar distance = 3.369 (2) and slippage = 1.945 (2) Å], and between the 4-bromo­phenyl rings of adjacent mol­ecules [centroid–centroid distance = 3.882 (2), inter­planar distance = 3.552 (2) and slippage = 1.566 (2) Å]. A Br⋯Br [3.6446 (4) Å] halogen inter­action is also observed.

## Related literature

For background information and the crystal structures of related compounds, see: Choi *et al.* (2008 ▶, 2010 ▶).
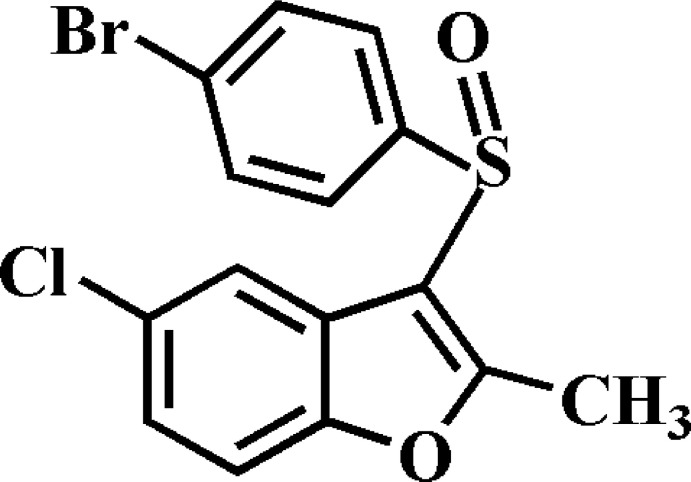


## Experimental

### 

#### Crystal data

C_15_H_10_BrClO_2_S
*M* *_r_* = 369.65
Triclinic, 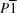

*a* = 6.4192 (1) Å
*b* = 9.9185 (2) Å
*c* = 11.7755 (2) Å
α = 100.681 (1)°
β = 92.113 (1)°
γ = 104.168 (1)°
*V* = 711.67 (2) Å^3^
*Z* = 2
Mo *K*α radiation
μ = 3.22 mm^−1^
*T* = 173 K
0.38 × 0.31 × 0.27 mm

#### Data collection

Bruker SMART APEXII CCD diffractometer
Absorption correction: multi-scan (*SADABS*; Bruker, 2009 ▶) *T*_min_ = 0.374, *T*_max_ = 0.475
13131 measured reflections
3529 independent reflections
3236 reflections with *I* > 2σ(*I*)
*R*_int_ = 0.034

#### Refinement

*R*[*F* ^2^ > 2σ(*F* ^2^)] = 0.031
*wR*(*F* ^2^) = 0.081
*S* = 1.12
3529 reflections
182 parameters
H-atom parameters constrained
Δρ_max_ = 0.55 e Å^−3^
Δρ_min_ = −0.76 e Å^−3^

### 

Data collection: *APEX2* (Bruker, 2009 ▶); cell refinement: *SAINT* (Bruker, 2009 ▶); data reduction: *SAINT*; program(s) used to solve structure: *SHELXS97* (Sheldrick, 2008 ▶); program(s) used to refine structure: *SHELXL97* (Sheldrick, 2008 ▶); molecular graphics: *ORTEP-3* (Farrugia, 1997 ▶) and *DIAMOND* (Brandenburg, 1998 ▶); software used to prepare material for publication: *SHELXL97*.

## Supplementary Material

Crystal structure: contains datablock(s) I. DOI: 10.1107/S1600536812025846/ds2198sup1.cif

Structure factors: contains datablock(s) I. DOI: 10.1107/S1600536812025846/ds2198Isup2.hkl

Supplementary material file. DOI: 10.1107/S1600536812025846/ds2198Isup3.cml

Additional supplementary materials:  crystallographic information; 3D view; checkCIF report

## Figures and Tables

**Fig. 1.**
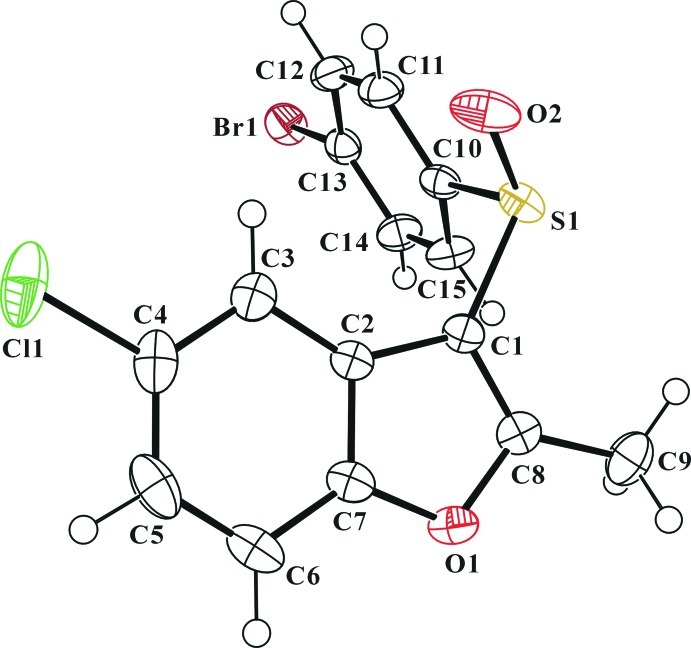
The molecular structure of the title compound with the atom numbering scheme. Displacement ellipsoids are drawn at the 50% probability level. H atoms are presented as small spheres of arbitrary radius.

**Fig. 2.**
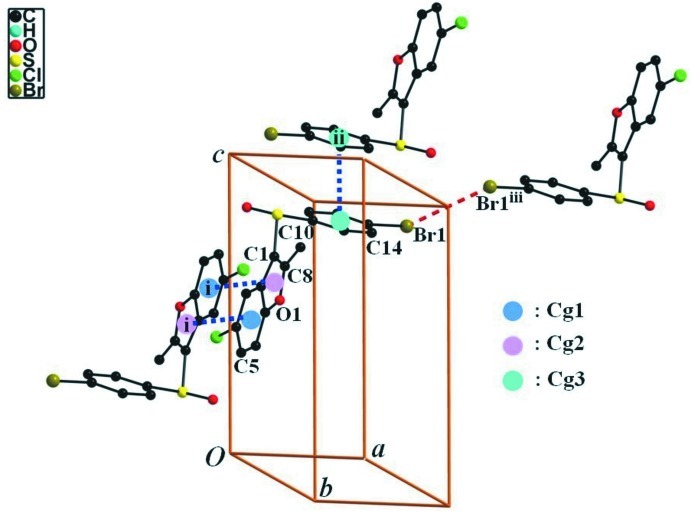
A view of the π···π and Br···Br interactions (dotted lines) in the crystal structure of the title compound. H atoms were omitted for clarity. [Symmetry codes: (i) - x, - y, - z + 1; (ii) - x + 1, - y + 1, - z + 2; (iii) - x + 2, - y + 2, - z + 2.]

## supplementary crystallographic information

### Comment

As a part of our ongoing study of 5-chloro-2-methyl-1-benzofuran derivatives containing 3-phenylsulfonyl (Choi *et al.*, 2008) and 3-(4-fluorophenylsulfonyl) (Choi *et al.*, 2010) substituents, we report herein the crystal structure of the title compound.

In the title molecule (Fig. 1), the benzofuran unit is essentially planar, with a mean deviation of 0.009 (2) Å from the least-squares plane defined by the nine constituent atoms. The dihedral angle between the 4-bromophenyl ring and the mean plane of the benzofuran fragment is 86.85 (6)°. In the crystal structure (Fig. 2), molecules are connected by slipped π···π interactions; the first one between the benzene and the furan rings of adjacent molecules, with a Cg1···Cg2^i^ distance of 3.884 (2) Å and an interplanar distance of 3.369 (2) Å resulting in a slippage of 1.945 (2) Å (Cg1 and Cg2 are the centroids of the C2–C7 benzene ring and the C1/C2/C7/O1/C8 furan ring, respectively), and the second one between the 4-bromophenyl rings of adjacent molecules, with a Cg3···Cg3^ii^ distance of 3.882 (2) Å and an interplanar distance of 3.552 (2) Å resulting in a slippage of 1.566 (2) Å (Cg3 is the centroid of the C10–C15 benzene ring). The crystal packing (Fig. 2) also exhibits a Br···Br contact at [3.6446 (4) Å].

### Experimental

3-Chloroperoxybenzoic acid (77%, 202 mg, 0.9 mmol) was added in small portions to a stirred solution of 3-(4-bromophenylsulfanyl)-5-chloro-2-methyl-1-benzofuran (283 mg, 0.8 mmol) in dichloromethane (30 mL) at 273 K. After being stirred at room temperature for 5h, the mixture was washed with saturated sodium bicarbonate solution and the organic layer was separated, dried over magnesium sulfate, filtered and concentrated at reduced pressure. The residue was purified by column chromatography (hexane/ethyl acetate, 2:1 v/v) to afford the title compound as a colorless solid [yield 73%, m.p. 395–396 K; *R*_f_ = 0.44 (hexane/ethyl acetate, 2:1 v/v)]. Single crystals suitable for X-ray diffraction were prepared by slow evaporation of a solution of the title compound in ethyl acetate at room temperature.

### Refinement

All H atoms were positioned geometrically and refined using a riding model, with C—H = 0.95 Å for aryl and 0.98 Å for methyl H atoms. Uiso(H) = 1.2*U*_eq_(C) for aryl and 1.5*U*_eq_(C) for methyl H atoms. The positions of methyl hydrogens were optimized rotationally.

### Crystal data

**Table:**

C_15_H_10_BrClO_2_S	*Z* = 2
*M**_r_* = 369.65	*F*(000) = 368
Triclinic, *P*1	*D*_x_ = 1.725 Mg m^−^^3^
Hall symbol: -P 1	Mo *K*α radiation, λ = 0.71073 Å
*a* = 6.4192 (1) Å	Cell parameters from 7922 reflections
*b* = 9.9185 (2) Å	θ = 2.5–28.3°
*c* = 11.7755 (2) Å	µ = 3.22 mm^−^^1^
α = 100.681 (1)°	*T* = 173 K
β = 92.113 (1)°	Block, colourless
γ = 104.168 (1)°	0.38 × 0.31 × 0.27 mm
*V* = 711.67 (2) Å^3^	

### Data collection

**Table:**

Bruker SMART APEXII CCD diffractometer	3529 independent reflections
Radiation source: rotating anode	3236 reflections with *I* > 2σ(*I*)
Graphite multilayer monochromator	*R*_int_ = 0.034
Detector resolution: 10.0 pixels mm^-1^	θ_max_ = 28.3°, θ_min_ = 1.8°
φ and ω scans	*h* = −8→8
Absorption correction: multi-scan (*SADABS*; Bruker, 2009)	*k* = −13→13
*T*_min_ = 0.374, *T*_max_ = 0.475	*l* = −15→15
13131 measured reflections	

### Refinement

**Table:**

Refinement on *F*^2^	Primary atom site location: structure-invariant direct methods
Least-squares matrix: full	Secondary atom site location: difference Fourier map
*R*[*F*^2^ > 2σ(*F*^2^)] = 0.031	Hydrogen site location: difference Fourier map
*wR*(*F*^2^) = 0.081	H-atom parameters constrained
*S* = 1.12	*w* = 1/[σ^2^(*F*_o_^2^) + (0.0418*P*)^2^ + 0.3308*P*] where *P* = (*F*_o_^2^ + 2*F*_c_^2^)/3
3529 reflections	(Δ/σ)_max_ = 0.001
182 parameters	Δρ_max_ = 0.55 e Å^−^^3^
0 restraints	Δρ_min_ = −0.76 e Å^−^^3^

### Special details

**Table:**

Geometry. All esds (except the esd in the dihedral angle between two l.s. planes) are estimated using the full covariance matrix. The cell esds are taken into account individually in the estimation of esds in distances, angles and torsion angles; correlations between esds in cell parameters are only used when they are defined by crystal symmetry. An approximate (isotropic) treatment of cell esds is used for estimating esds involving l.s. planes.
Refinement. Refinement of F^2^ against ALL reflections. The weighted R-factor wR and goodness of fit S are based on F^2^, conventional R-factors R are based on F, with F set to zero for negative F^2^. The threshold expression of F^2^ > 2sigma(F^2^) is used only for calculating R-factors(gt) etc. and is not relevant to the choice of reflections for refinement. R-factors based on F^2^ are statistically about twice as large as those based on F, and R- factors based on ALL data will be even larger.

### Fractional atomic coordinates and isotropic or equivalent isotropic displacement parameters (Å^2^)

**Table:**

	*x*	*y*	*z*	*U*_iso_*/*U*_eq_	
Br1	0.73945 (4)	0.92053 (2)	0.917091 (18)	0.03412 (8)	
Cl1	−0.34796 (9)	0.39254 (7)	0.44410 (7)	0.05267 (19)	
S1	0.18432 (8)	0.27154 (5)	0.84285 (4)	0.02666 (11)	
O1	0.3068 (2)	0.10245 (15)	0.53223 (12)	0.0280 (3)	
O2	−0.0436 (3)	0.27377 (19)	0.86385 (15)	0.0408 (4)	
C1	0.1970 (3)	0.21347 (19)	0.69398 (16)	0.0221 (3)	
C2	0.0811 (3)	0.23994 (19)	0.59651 (16)	0.0218 (3)	
C3	−0.0758 (3)	0.3123 (2)	0.58146 (19)	0.0273 (4)	
H3	−0.1307	0.3616	0.6456	0.033*	
C4	−0.1474 (3)	0.3084 (2)	0.4682 (2)	0.0329 (5)	
C5	−0.0676 (4)	0.2384 (3)	0.3725 (2)	0.0381 (5)	
H5	−0.1203	0.2402	0.2965	0.046*	
C6	0.0869 (4)	0.1664 (3)	0.38686 (19)	0.0353 (5)	
H6	0.1427	0.1179	0.3225	0.042*	
C7	0.1560 (3)	0.1686 (2)	0.49963 (17)	0.0254 (4)	
C8	0.3276 (3)	0.1306 (2)	0.65100 (18)	0.0253 (4)	
C9	0.4833 (4)	0.0697 (2)	0.7071 (2)	0.0357 (5)	
H9A	0.6304	0.1255	0.7026	0.054*	
H9B	0.4668	−0.0287	0.6672	0.054*	
H9C	0.4555	0.0720	0.7886	0.054*	
C10	0.3331 (3)	0.4532 (2)	0.85442 (15)	0.0222 (3)	
C11	0.2396 (3)	0.5607 (2)	0.89910 (18)	0.0283 (4)	
H11	0.0936	0.5387	0.9172	0.034*	
C12	0.3588 (3)	0.7010 (2)	0.91761 (18)	0.0296 (4)	
H12	0.2961	0.7758	0.9486	0.036*	
C13	0.5709 (3)	0.7301 (2)	0.89001 (16)	0.0247 (4)	
C14	0.6662 (3)	0.6227 (2)	0.84636 (19)	0.0294 (4)	
H14	0.8119	0.6448	0.8279	0.035*	
C15	0.5474 (3)	0.4829 (2)	0.82977 (19)	0.0292 (4)	
H15	0.6119	0.4080	0.8018	0.035*	

### Atomic displacement parameters (Å^2^)

**Table:**

	*U*^11^	*U*^22^	*U*^33^	*U*^12^	*U*^13^	*U*^23^
Br1	0.04322 (14)	0.02544 (12)	0.02911 (12)	0.00304 (9)	0.00126 (9)	0.00208 (8)
Cl1	0.0273 (3)	0.0545 (4)	0.0839 (5)	0.0083 (2)	−0.0072 (3)	0.0384 (4)
S1	0.0281 (2)	0.0293 (2)	0.0188 (2)	0.00123 (18)	0.00349 (17)	0.00343 (18)
O1	0.0258 (7)	0.0302 (7)	0.0268 (7)	0.0092 (6)	0.0044 (5)	−0.0010 (6)
O2	0.0277 (8)	0.0484 (9)	0.0358 (8)	−0.0032 (7)	0.0136 (6)	−0.0022 (7)
C1	0.0210 (8)	0.0223 (8)	0.0210 (8)	0.0032 (7)	0.0012 (7)	0.0024 (7)
C2	0.0194 (8)	0.0224 (8)	0.0214 (8)	0.0020 (7)	0.0022 (7)	0.0032 (7)
C3	0.0207 (9)	0.0270 (9)	0.0344 (10)	0.0038 (7)	0.0034 (7)	0.0090 (8)
C4	0.0203 (9)	0.0330 (10)	0.0460 (12)	0.0002 (8)	−0.0024 (8)	0.0195 (9)
C5	0.0302 (11)	0.0500 (13)	0.0286 (10)	−0.0052 (9)	−0.0063 (8)	0.0165 (10)
C6	0.0338 (11)	0.0448 (12)	0.0215 (9)	0.0014 (9)	0.0033 (8)	0.0035 (9)
C7	0.0208 (8)	0.0290 (9)	0.0233 (9)	0.0024 (7)	0.0026 (7)	0.0025 (7)
C8	0.0220 (9)	0.0233 (8)	0.0283 (9)	0.0030 (7)	0.0014 (7)	0.0032 (7)
C9	0.0286 (10)	0.0325 (11)	0.0471 (13)	0.0110 (8)	−0.0020 (9)	0.0075 (10)
C10	0.0216 (8)	0.0264 (9)	0.0160 (8)	0.0043 (7)	0.0001 (6)	0.0002 (7)
C11	0.0218 (9)	0.0363 (10)	0.0279 (10)	0.0105 (8)	0.0053 (7)	0.0042 (8)
C12	0.0308 (10)	0.0311 (10)	0.0290 (10)	0.0150 (8)	0.0053 (8)	0.0014 (8)
C13	0.0287 (9)	0.0246 (9)	0.0189 (8)	0.0057 (7)	−0.0013 (7)	0.0022 (7)
C14	0.0215 (9)	0.0316 (10)	0.0337 (10)	0.0067 (8)	0.0054 (8)	0.0026 (8)
C15	0.0251 (9)	0.0290 (10)	0.0323 (10)	0.0090 (8)	0.0087 (8)	−0.0005 (8)

### Geometric parameters (Å, º)

**Table:**

Br1—C13	1.895 (2)	C5—H5	0.9500
Br1—Br1^i^	3.6446 (4)	C6—C7	1.379 (3)
Cl1—C4	1.738 (2)	C6—H6	0.9500
Cl1—Cl1^ii^	3.3576 (15)	C8—C9	1.482 (3)
S1—O2	1.4973 (16)	C9—H9A	0.9800
S1—C1	1.7522 (19)	C9—H9B	0.9800
S1—C10	1.798 (2)	C9—H9C	0.9800
O1—C8	1.369 (2)	C10—C11	1.380 (3)
O1—C7	1.375 (2)	C10—C15	1.388 (3)
C1—C8	1.360 (3)	C11—C12	1.387 (3)
C1—C2	1.444 (3)	C11—H11	0.9500
C2—C7	1.393 (3)	C12—C13	1.385 (3)
C2—C3	1.396 (3)	C12—H12	0.9500
C3—C4	1.386 (3)	C13—C14	1.383 (3)
C3—H3	0.9500	C14—C15	1.383 (3)
C4—C5	1.392 (3)	C14—H14	0.9500
C5—C6	1.378 (4)	C15—H15	0.9500
			
C13—Br1—Br1^i^	129.17 (6)	C1—C8—O1	110.68 (17)
C4—Cl1—Cl1^ii^	147.84 (10)	C1—C8—C9	132.7 (2)
O2—S1—C1	108.52 (9)	O1—C8—C9	116.58 (17)
O2—S1—C10	106.57 (9)	C8—C9—H9A	109.5
C1—S1—C10	98.01 (9)	C8—C9—H9B	109.5
C8—O1—C7	106.55 (14)	H9A—C9—H9B	109.5
C8—C1—C2	107.50 (16)	C8—C9—H9C	109.5
C8—C1—S1	123.01 (15)	H9A—C9—H9C	109.5
C2—C1—S1	129.49 (14)	H9B—C9—H9C	109.5
C7—C2—C3	119.56 (18)	C11—C10—C15	120.93 (18)
C7—C2—C1	104.41 (16)	C11—C10—S1	119.38 (15)
C3—C2—C1	136.02 (18)	C15—C10—S1	119.34 (15)
C4—C3—C2	116.62 (19)	C10—C11—C12	119.95 (18)
C4—C3—H3	121.7	C10—C11—H11	120.0
C2—C3—H3	121.7	C12—C11—H11	120.0
C3—C4—C5	122.8 (2)	C13—C12—C11	118.80 (18)
C3—C4—Cl1	118.67 (18)	C13—C12—H12	120.6
C5—C4—Cl1	118.47 (17)	C11—C12—H12	120.6
C6—C5—C4	120.8 (2)	C14—C13—C12	121.50 (19)
C6—C5—H5	119.6	C14—C13—Br1	118.71 (15)
C4—C5—H5	119.6	C12—C13—Br1	119.75 (15)
C5—C6—C7	116.4 (2)	C13—C14—C15	119.41 (18)
C5—C6—H6	121.8	C13—C14—H14	120.3
C7—C6—H6	121.8	C15—C14—H14	120.3
O1—C7—C6	125.35 (18)	C14—C15—C10	119.37 (18)
O1—C7—C2	110.85 (17)	C14—C15—H15	120.3
C6—C7—C2	123.8 (2)	C10—C15—H15	120.3
			
O2—S1—C1—C8	−144.10 (17)	C1—C2—C7—C6	−179.82 (19)
C10—S1—C1—C8	105.36 (17)	C2—C1—C8—O1	0.7 (2)
O2—S1—C1—C2	35.8 (2)	S1—C1—C8—O1	−179.37 (13)
C10—S1—C1—C2	−74.70 (18)	C2—C1—C8—C9	179.8 (2)
C8—C1—C2—C7	−0.4 (2)	S1—C1—C8—C9	−0.3 (3)
S1—C1—C2—C7	179.69 (15)	C7—O1—C8—C1	−0.7 (2)
C8—C1—C2—C3	178.4 (2)	C7—O1—C8—C9	−179.98 (17)
S1—C1—C2—C3	−1.6 (3)	O2—S1—C10—C11	11.79 (19)
C7—C2—C3—C4	−0.2 (3)	C1—S1—C10—C11	123.91 (16)
C1—C2—C3—C4	−178.8 (2)	O2—S1—C10—C15	−174.93 (16)
C2—C3—C4—C5	−0.9 (3)	C1—S1—C10—C15	−62.81 (18)
C2—C3—C4—Cl1	178.31 (14)	C15—C10—C11—C12	1.4 (3)
Cl1^ii^—Cl1—C4—C3	8.9 (3)	S1—C10—C11—C12	174.58 (16)
Cl1^ii^—Cl1—C4—C5	−171.87 (13)	C10—C11—C12—C13	0.3 (3)
C3—C4—C5—C6	1.1 (3)	C11—C12—C13—C14	−1.0 (3)
Cl1—C4—C5—C6	−178.09 (17)	C11—C12—C13—Br1	−178.79 (15)
C4—C5—C6—C7	−0.2 (3)	Br1^i^—Br1—C13—C14	−52.56 (18)
C8—O1—C7—C6	−179.8 (2)	Br1^i^—Br1—C13—C12	125.32 (15)
C8—O1—C7—C2	0.5 (2)	C12—C13—C14—C15	0.0 (3)
C5—C6—C7—O1	179.31 (19)	Br1—C13—C14—C15	177.84 (16)
C5—C6—C7—C2	−1.0 (3)	C13—C14—C15—C10	1.7 (3)
C3—C2—C7—O1	−179.05 (16)	C11—C10—C15—C14	−2.4 (3)
C1—C2—C7—O1	−0.1 (2)	S1—C10—C15—C14	−175.55 (16)
C3—C2—C7—C6	1.2 (3)		

Symmetry codes: (i) −*x*+2, −*y*+2, −*z*+2; (ii) −*x*−1, −*y*+1, −*z*+1.
